# Chromatic Pupillometry as a Putative Screening Tool for Heritable Retinal Disease in Rhesus Macaques

**DOI:** 10.1167/tvst.12.6.13

**Published:** 2023-06-22

**Authors:** Elyse M. Salpeter, Ala Moshiri, Michelle Ferneding, Monica J. Motta, Sangwan Park, Chrisoula Skouritakis, Sara M. Thomasy

**Affiliations:** 1 Department of Surgical and Radiological Sciences, School of Veterinary Medicine, University of California Davis, Davis, CA, USA; 2 Department of Ophthalmology and Vision Science, School of Medicine, University of California Davis, Davis, CA, USA

**Keywords:** inherited retinal diseases, dark adaptation, chromatic pupillometry, achromatopsia, non-human primates

## Abstract

**Purpose:**

Non-human primates (NHPs) are useful models for human retinal disease. Chromatic pupillometry has been proposed as a noninvasive method of identifying inherited retinal diseases (IRDs) in humans; however, standard protocols employ time-consuming dark adaptation. We utilized shortened and standard dark-adaptation protocols to compare pupillary light reflex characteristics following chromatic stimulation in rhesus macaques with achromatopsia to wild-type (WT) controls with normal retinal function.

**Methods:**

Nine rhesus macaques homozygous for the p.R656Q mutation (*PDE6C* HOMs) and nine WT controls were evaluated using chromatic pupillometry following 1-minute versus standard 20-minute dark adaptations. The following outcomes were measured and compared between groups: pupil constriction latency, peak constriction, pupil constriction time, and constriction velocity.

**Results:**

Pupil constriction latency was significantly longer in *PDE6C* HOMs with red-light (*P* = 0.0002) and blue-light (*P* = 0.04) stimulation versus WT controls. Peak constriction was significantly less in *PDE6C* HOMs with all light stimulation compared to WT controls (*P* < 0.0001). Pupil constriction time was significantly shorter in *PDE6C* HOMs versus WT controls with red-light (*P* = 0.04) and white-light (*P* = 0.003) stimulation. Pupil constriction velocity was significantly slower in *PDE6C* HOMs versus WT controls with red-light (*P* < 0.0001), blue-light (*P* < 0.0001), and white-light (*P* = 0.0002) stimulation. Dark adaptation time only significantly affected peak (*P* = 0.008) and time of pupil constriction (*P* = 0.02) following blue-light stimulation.

**Conclusions:**

Chromatic pupillometry following 1- and 20-minute dark adaptation is an effective tool for screening NHPs for achromatopsia.

**Translational Relevance:**

Rapid identification of NHPs with IRDs will provide animal research models to advance research and treatment of achromatopia in humans.

## Introduction

Inherited retinal diseases (IRDs) encompass a group of disorders with a genetic etiology that affect various cells and layers of the retina, resulting in severe vision impairment or blindness. Some common IRDs affecting humans include Leber congenital amaurosis (LCA), retinitis pigmentosa, Stargardt's disease, and achromatopsia (ACHM), with incidence rates of approximately 1 in 3300,^1^ 1 in 3500,^2^ 1 in 10,000,^3^ and 1 in 10,000,^4^ respectively. These diseases share a common pathophysiology involving photoreceptor cells, although ACHM is predominantly a cone disorder and the others have cone and rod involvement.^1^^–^^4^ Currently, the only U.S. Food and Drug Administration–approved treatment for any of these disorders is gene therapy, which is limited to a biallelic *RPE65* mutation causing LCA and retinitis pigmentosa.^5^

Appropriate animal models that replicate human disease are required for further therapeutic development for IRDs. Currently, most retinal disease research employs murine models of physiology and pathology due to the relative ease of genetic manipulation and quick breeding. Despite this, the utility of mice as animal models for retinal disease in humans is restricted due to differences in cytoarchitecture, synaptic connections, retinal vascular bed, retinal aging, photoreceptor adaptations, and a complete absence of the macula.^6^^–^^10^ The consequence of these differences is reflected in a study by Coleman et al.,^11^ wherein deletion of the guanylate cyclase 2D (*GUCY2D*) gene in mice resulted in cone-specific photoreceptor degradation only, in contrast to the complete photoreceptor degeneration noted in humans with LCA1 caused by a sporadic mutation in the same gene. By contrast, non-human primates (NHPs) are more ideal animal models to investigate retinal diseases, particularly cone disorders, due to strong resemblances in retinal anatomy, particularly the fovea. ACHM due to a spontaneous missense mutation in the catalytic region of the alpha subunit of cone-specific phosphodiesterase 6 (*PDE6C*) has been recently described in rhesus macaques.^12^

Despite their utility in advancing biomedical research, the use of NHP models is often limited by low prevalence of spontaneous disease in research colonies, often leading to missed disease phenotypes.^13^ Furthermore, individual examination and testing are extremely time consuming, expensive, and limited by current biological and social factors within the colony. To overcome these limitations, spontaneous disease detection in large colonies relies on annual or biannual screening events colloquially termed “round-up.” The high-throughput, time-restricted nature of round-up events demands rapid and simple screening tools to identify spontaneous diseases of interest. Currently, detection of NHP models of IRDs relies on a multitude of diagnostics such as clinical examination, advanced ocular imaging, and electroretinography. The expensive, time-consuming nature of these tests prevents their use for disease screening, particularly during round-up events. A simple, non-invasive screening tool is required to facilitate easier identification of NHPs with IRDs relevant to human disease.

Significant changes in pupillary responses to chromatic light stimulation have been identified in humans with a variety of IRDs, most notably retinitis pigmentosa, LCA, and ACHM.^14^^–^^20^ The decreased expense and ease of use of chromatic pupillometry in comparison to classic retinal testing have stimulated interest in using this tool for diagnosis and progression monitoring of IRDs in humans. Chromatic pupillometry facilitates assessment of various retinal cells involved in the pupillary light reflex (PLR) pathway through the use of two different light wavelengths. Blue-light stimulation (480 nm) selectively activates rods, short-wavelength cones, and intrinsically photosensitive retinal ganglion cells (ipRGCs).^21^^–^^24^ Comparatively, red-light stimulus (630 nm) is outside the spectral sensitivity of ipRGCs and therefore provides clinical approximation of cone photoreceptor function. The neurobiological principles of chromatic pupillometry and aforementioned documentation of chromatic PLR changes in humans with various IRDs suggest that chromatic pupillometry may serve as a simple, inexpensive test to identify animal models of IRDs.

Despite the promising potential of chromatic pupillometry for cost-effective, simple identification of IRD in NHPs, current dark adaptation protocols are too time consuming to be of use for screening during round-up events. Previous reports investigating chromatic pupillometry in humans with IRDs most commonly employed dark adaptation protocols 10 minutes in length prior to PLR stimulation.^14^^,^^15^^,^^17^^,^^20^ Other studies extended dark adaptation to 20 to 30 minutes to ensure adequate retinal sensitivity.^16^^,^^19^ Investigation of dark-adaptation–induced changes in rod, cone, and ipRGC sensitivity supports a 20-minute dark adaptation prior to PLR testing to achieve consistent, stable pupil responses,^25^ but a consensus protocol has not been established. Furthermore, chromatic pupillometry has not been studied in NHPs.

The purpose of this study was to assess the feasibility of chromatic pupillometry as a screening tool following a standard and shortened dark-adaptation protocol in rhesus macaques with *PDE6C-*associated ACHM versus wild-type (WT) controls with normal retinal function. The authors hypothesized the following: (1) pupillary responses to chromatic light stimulation would vary significantly between homozygotes (HOMs) and WT controls, most notably following red-light stimuli; and (2) a shortened dark-adaptation protocol would be sufficient for detecting significant differences in pupillary responses to chromatic light stimulation between groups.

## Materials and Methods

### Study Animals

All rhesus macaques in this study underwent complete ophthalmic examinations at the California National Primate Research Center (CNPRC). Study protocols at CNPRC followed guidelines of the ARVO Statement for the Use of Animals in Ophthalmic and Vision Research, complied with National Institutes of Health Guide for the Care and Use of Laboratory Animals, and were approved by the University of California, Davis Institutional Animal Care and Use Committee.

### Session 1: Baseline Data From General Population

Sixteen rhesus macaques from the general population were randomly selected to establish baseline chromatic pupillometry data. Sanger sequencing determined that all primates were WT for the *PDE6C* gene. Phenotypic status was confirmed by the following ophthalmic testing protocol: Macaques were sedated with intramuscular (IM) injections of ketamine (5–30 mg/kg IM), midazolam (0.1 mg/kg IM), and dexmedetomidine (0.05–0.075 mg/kg IM). Mydriasis was achieved with tropicamide (1%; Bausch & Lomb, Bridgewater, NJ) and phenylephrine (2.5%; Paragon Biosciences, Northbrook, IL), and cycloplegia was achieved with cyclopentolate (1%; Akorn, Lake Forest, IL). All macaques underwent a comprehensive eye examination including portable slit-lamp examination, indirect ophthalmoscopy, rebound tonometry (TonoVet; Icare, Vantaa, Finland), spectral-domain optical coherence tomography (SD-OCT) with confocal scanning laser ophthalmoscopy (SPECTRALIS; Heidelberg Engineering, Heidelberg, Germany), and full-field scotopic and photopic electroretinography (RetEvet handheld unit; LKC Technologies, Gaithersberg, MD). A 30° horizontal high-resolution raster 127 scan centered on the fovea was obtained using a corneal curvature (K) value of 6.5-mm radius for each SPECTRALIS image. An artificial tear solution (GenTeal; Alcon, Geneva, Switzerland) was used to maintain the ocular surface during the entirety of imaging. When normal phenotypic status had been confirmed, chromatic pupillometry was performed on a separate testing day.

Rhesus macaques with ACHM were initially identified through Sanger sequencing for a homozygous missense mutation of the R565Q mutation in PDE6C (*PDE6C* HOMs) as previously described.^12^ Age- and sex-matched WT controls with normal vision were selected. Phenotypic status consistent with ACHM was confirmed using the same ophthalmic testing protocol described above. The thicknesses of the total retina, inner photoreceptor segments, and outer photoreceptor segments were manually measured on SD-OCT images at the foveal center and at a distance of 1.5 mm from each side of the fovea using calipers in ImageJ software (National Institutes of Health, Bethesda, MD) according to established guidelines.^26^

### Chromatic Pupillometry

To acquire baseline pupillometry data, 16 phenotypically normal WT rhesus macaques were dark adapted in a room free from any light sources for 20 minutes. After dark adaptation, an eyelid speculum was placed oculus sinister (OS) or oculus dexter (OD), as determined randomly. Pupillary light reflexes were elicited using red-light (630 nm), blue-light (480 nm), and white-light–emitting diode (LED) light sources using the Melan-100 Pupil Light Reflex Stimulator (BioMed Vision Technologies, Ames, IA) with a light intensity of 200 kcd/m^2^ and held 1 inch from the corneal surface (according to manufacturer's instructions for use in humans) in the dark room. For the first eye, direct red-, blue-, and then white-light stimulation was applied for 13 seconds each, separated by 60 seconds of dark adaptation. The eye not being tested was gently held closed with 1-inch micropore surgical tape (Micropore Surgical Tape 1530; 3M, St. Paul, MN). This light stimulation protocol was then repeated for the other eye after 60 seconds.

### Session 2: Comparative Study Between HOM *PDE6C* and WT Controls

**Figure 1.**
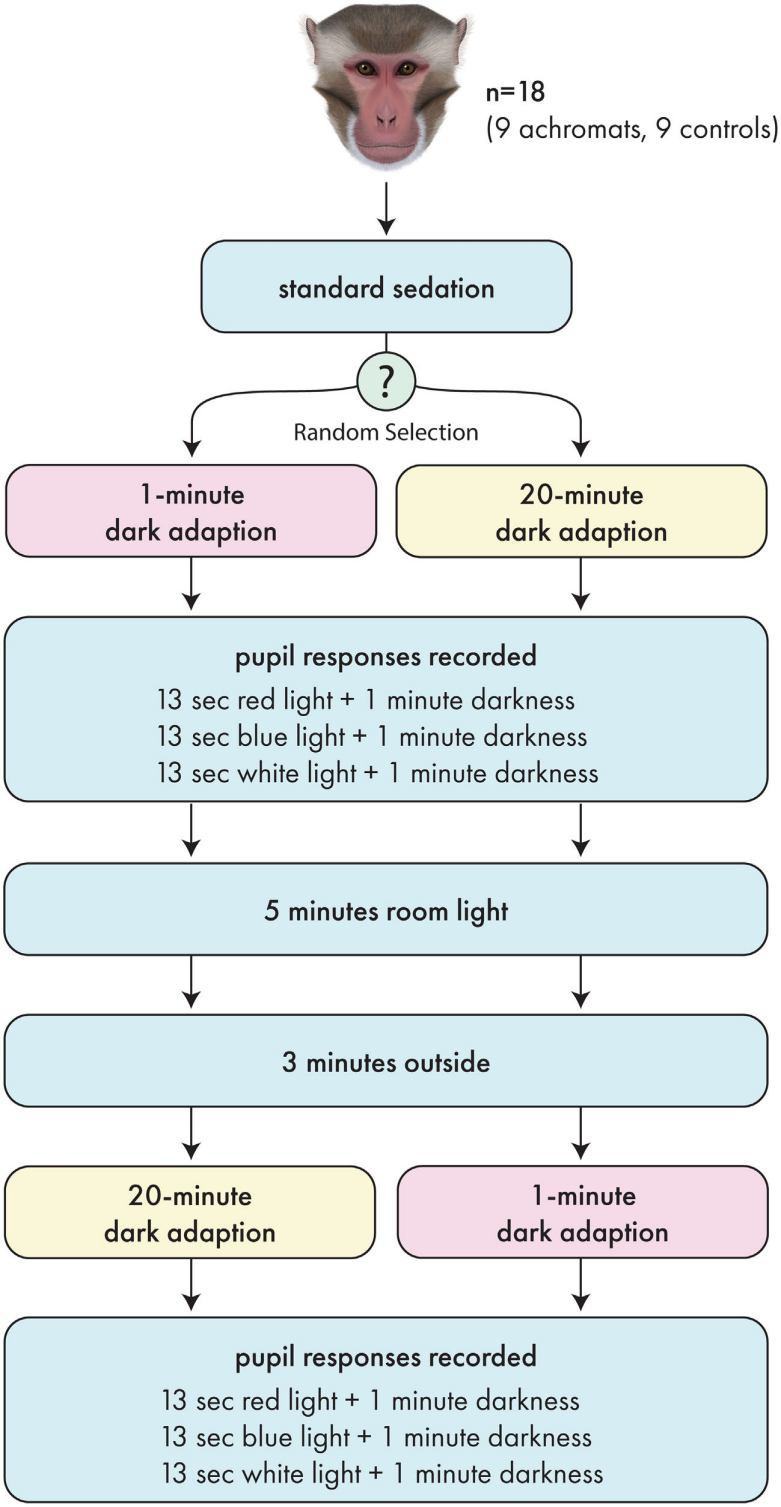
Chromatic pupillometry protocol for measuring pupil responses to red, blue, and white light in WT controls and *PDE6C* HOMs following a 1- or 20-minute dark-adaptation protocol.

**Figure 2.**
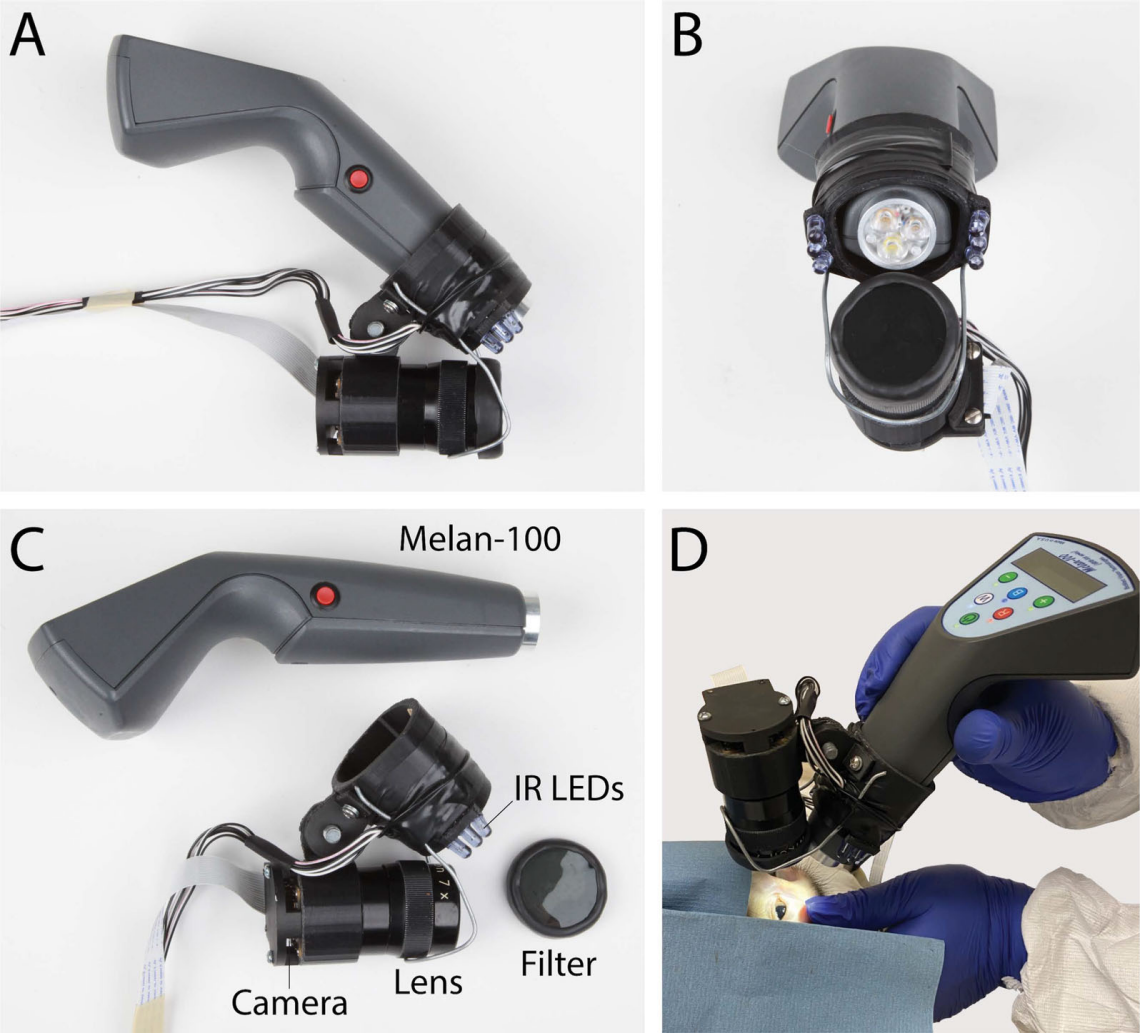
Melan-100 with video recording device fitted with a coaxial infrared video retinoscopic attachment. Attachment of the video retinoscope on Melan-100 viewed from the *side* (**A**) and *front* (**B**); separate components of Melan-100 and video retinoscopic attachment (**C**). Representative image of pupillometry testing in normal rhesus macaque using Melan-100 with video retinoscopic attachment (**D**).

Following successful acquisition of baseline pupillometry data from normal rhesus macaques in the general population, chromatic pupillometry was completed twice within the same day on the *PDE6C* HOMs and WT controls using a protocol outlined in Figure 1. The direct PLR during 13 seconds of exposure to serial red, blue, and white light was recorded once for each eye following dark-adaptation protocols using a video recording (Fig. 2). The video recording device consisted of a 60-mm Nikkor lens (F-2.8; Nikon, Melville, NY) fitted with a coaxial infrared video retinoscopic attachment (Raspberry Pi, 1080 pixels; Pencoed, Wales, UK) attached to a video monitor. Infrared LEDs were mounted on either side of a three-dimensional printed sleeve through which the Melan-100 device was inserted in order to provide illumination for video capture of the resting pupil size without inadvertent retinal stimulation.

### Pupil Measurements

ImageJ was used to measure and calculate the following study outcomes from the captured video recordings of each eye:

• *Latency*—Time from stimulus onset until start of pupil constriction
• *Peak pupil constriction*—Percentage change in pupil area calculated as:
  $$\begin{array}{c} \frac{\begin{array}{c} \mathrm{Baseline}\mathrm{pupil}\mathrm{area}-\mathrm{Minimum}\mathrm{pupil}\mathrm{area}\\ \;\mathrm{following}\mathrm{light}\mathrm{stimulation} \end{array}}{\mathrm{Baseline}\mathrm{pupil}\mathrm{area}}\times 100 \end{array}$$
• *Constriction time*—Period from the beginning of pupil constriction to time that minimum pupil size was achieved
• *Average constriction velocity*—Calculated as peak pupil constriction divided by the constriction time.

ImageJ was also used to measure and calculate consecutive pupil diameters every 2 seconds for a representative *PDE6C* HOMs and WT controls to facilitate the creation of representative PLR waveforms for all stimuli following both light-adaptation conditions. Relative amplitude was calculated as follows:

$$\begin{array}{c} \mathrm{RA}\left[\%\right]=\frac{\begin{array}{c} \mathrm{Baseline}\mathrm{pupil}\mathrm{area}-\mathrm{Pupil}\mathrm{area}\mathrm{following}\\ \;\mathrm{light}\mathrm{stimulation}\mathrm{at}\mathrm{measurement}\\ \;timepoint\times 100 \end{array}}{\mathrm{Baseline}\mathrm{pupil}\mathrm{area}} \end{array}$$

### Statistics

A two-way, mixed-effects analysis of variance was used to assess differences in latency, peak, time, and average velocity of pupil constriction for each colored light stimulus among groups (PDE6C^R565Q^ HOMs vs. controls) and each dark-adaptation protocol. Additionally, a Pearson correlation was performed to assess the effect of age and sex on measured study outcomes averaged between both dark adaptation protocols for each color stimulant. Finally, a Pearson correlation was performed to assess the effect of amount of sedation required during testing, *PDE6C* status, and resting pupil size before PLR stimulation. Data were downloaded and managed in Excel (Microsoft Corporation, Redmond, WA). Statistical analyses were performed using Prism 8 (GraphPad, San Diego, CA). *P* < 0.05 was considered significant in all analyses. Finally, a receiver operating characteristic (ROC) curve analysis was performed for each pupil response parameter to evaluate the accuracy of each light stimulus to identify *PDE6C* HOMs among WT controls following the 1- and 20-minute dark adaptation. An area under the curve (AUC) > 0.8 was considered excellent, 0.7 to 0.8 was considered acceptable, and close to 0.5 was considered non-diagnostic in accordance with diagnostic scores and cut-off values previously established by Mandrekar.^27^

## Results

### Baseline Data From General Population

The ages of the evaluated individuals ranged from 0.8 to 22 years (mean ± SD, 10.8 ± 7), with an over-representation of females (12/16) due to their relative abundance in the overall colony population. Chromatic pupillometry data are summarized for the 16 rhesus macaques in Table 1.

**Table 1.** Chromatic Pupillometry Values for 12 Female and Four Male Clinically Normal Adult Rhesus Macaques Aged 0.8 to 22 Years Old

	Pupillary Light Reflex Characteristics			
Stimulus	Latency (ms)	Peak Constriction (%)	Constriction Time (ms)	Constriction Velocity (%/ms)
Red light	173.1 ± 31.1	54.8 ± 10.2	379.5 ± 117.4	0.1 ± 0.03
Blue light	192.7 ± 35.6	49.7 ± 7.9	383.1 ± 68.8	0.1 ± 0.03
White light	201.3 ± 18.6	50.4 ± 1.6	401.3 ± 40.3	0.1 ± 0.01

### Multimodal Ocular Imaging and Electroretinography of Study Population

**Figure 3.**
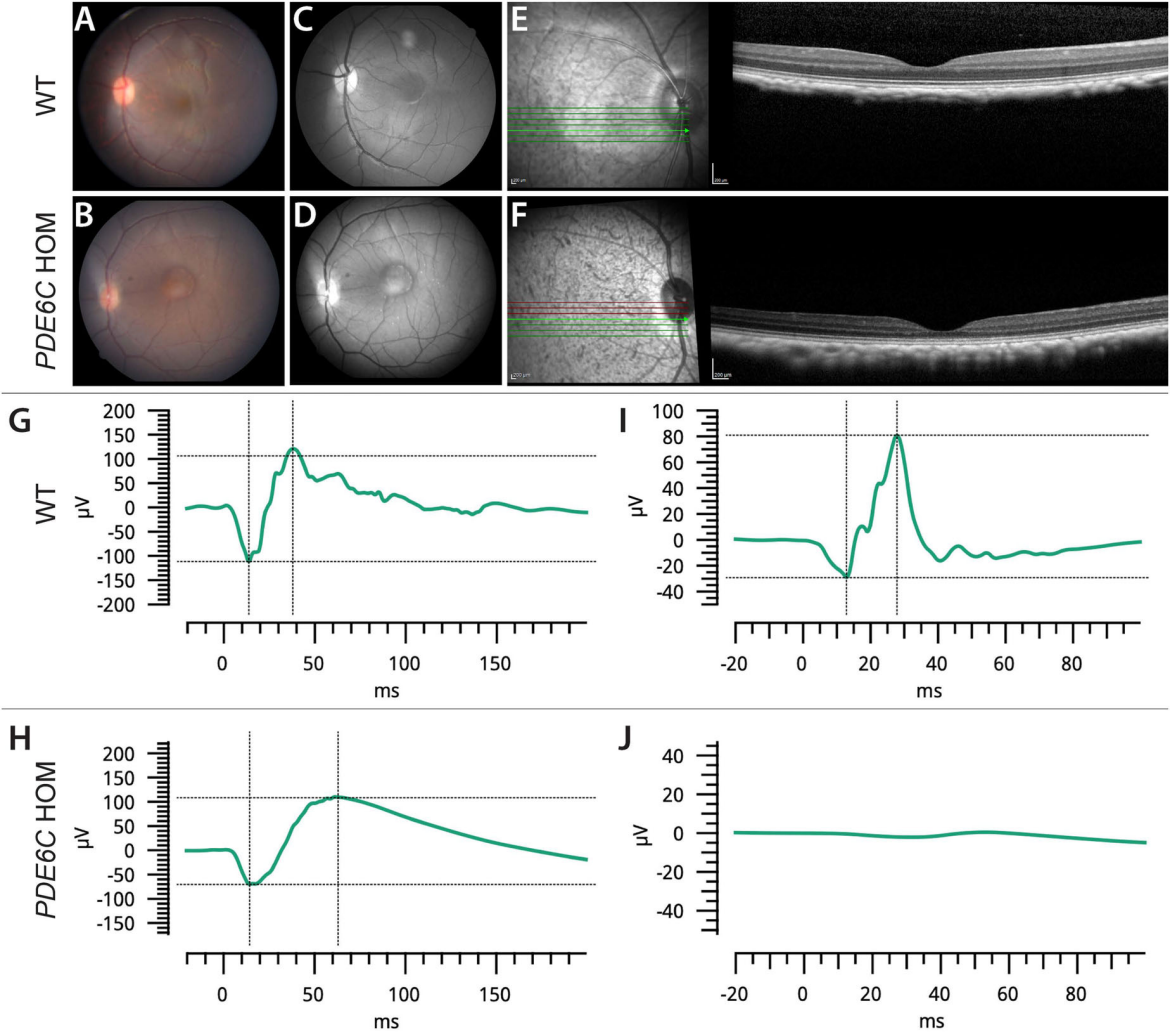
Multimodal retinal imaging and electroretinogram (ERG) of *PDE6C* HOMs demonstrate evidence of macular changes absent photopic retinal function consistent with ACHM. Color fundus photography (**A**, **B**) and red-free fundus photos (**C**, **D**) were obtained in *PDE6C* HOMs and WT controls. An example of a 3-year-old female WT rhesus macaque is shown (**A**, **C**), demonstrating normal fundus findings. The fundus photo of a 4-year-old female is reflective of changes noted in all *PDE6C* HOMs and shows a largely normal macular appearance, but with prominent foveal pigmentation (**B**, **D**). Representative OCT images from the same WT rhesus macaque (**E**) and *PDE6C* HOM (**F**) were acquired that demonstrated markedly reduced total foveal thickness in the *PDE6C* HOMs versus WT controls. Significant outer nuclear and outer plexiform layer thinning was noted in *PDE6C* HOMs versus WT controls in the temporal and nasal parafoveal region as noted in (**E**). (**G**, **I**) Representative ERG tracings were obtained from a WT control displaying normal a- and b-waves in scotopic (**G**) and photopic (**I**) conditions. (**H**, **J**) The *PDE6C* HOM (**H**, **J**) displayed normal scotopic waveforms in scotopic conditions (**H**) following a 3.0-cd·s/m^2^ stimulus but had absent waveforms in response to a 3.0-cd·s/m^2^ stimulus (**J**) following light adaptation.

Nine *PDE6C* HOMs (seven females and two males) and nine age- and sex-matched WT controls were included. Mean ages of the rhesus macaques with and without ACHM were 3.7 ± 1.4 and 4.2 ± 1.7 years, respectively. The *PDE6C* HOMs had mild foveal hyperpigmentation on fundic examination, foveal autofluorescence abnormalities with blue-light autofluorescence, and significant lateral, central, and nasal total retinal thinning due to photoreceptor thinning, as noted on SD-OCT versus WT controls (Fig. 3, Table 2). Punctate macular lesions, a common finding in macaques in our and other colonies,^28^^–^^33^ were noted on fundic examination in two of nine *PDE6C* HOMs and two of nine WT controls. No other fundus abnormalities were noted in the WT controls. Photopic responses with electroretinography were absent in all rhesus macaques with ACHM and present in the controls (Fig. 3), as previously described for rhesus macaques with *PDE6C* associated ACHM.^12^

**Table 2.** Total Retinal Thickness and Photoreceptor Inner and Outer Segment Thickness Were Significantly Reduced in *PDE6C* HOMs Versus Age- and Sex-Matched WT Controls

Measurement	Location	Group	Mean Thickness (µm) ± SE	*P*
Whole retina	Temporal	WT controls	314 ± 9.2	1.6 E^−4^*
		*PDE6C* HOMs	255 ± 9.2	
	Center	WT controls	193 ± 9.6	4.7 E^−5^*
		*PDE6C* HOMs	130 ± 9.6	
	Nasal	WT controls	325 ± 9.2	2.3 E^−4^*
		*PDE6C* HOMs	267 ± 9.2	
Photoreceptor outer segments	Temporal	WT controls	35.1 ± 1.4	1.0 E^−4^*
		*PDE6C* HOMs	26.2 ± 1.4	
	Center	WT controls	42.4 ± 2.3	4.6 E^−5^*
		*PDE6C* HOMs	27 ± 2.3	
	Nasal	WT controls	36.8 ± 1.7	1.9 E^−4^*
		*PDE6C* HOMs	26.0 ± 1.7	
Photoreceptor inner segments	Temporal	WT controls	28.3 ± 1.1	1.2 E^−7^*
		*PDE6C* HOMs	20.0 ± 1.1	
	Center	WT controls	30.3 ± 1.3	2.5 E^−6^*
		*PDE6C* HOMs	21.0 ± 1.3	
	Nasal	WT controls	29.4 ± 1.3	2.2 E^−6^*
		*PDE6C* HOMs	19.9 ± 1.3	
Nerve fiber layer	Temporal	WT controls	18.1 ± 1.0	0.2
		*PDE6C* HOMs	16.6 ± 1.0	
	Center	WT controls	18.2 ± 1.5	0.7 E^−1^
		*PDE6C* HOMs	15.2 ± 1.5	
	Nasal	WT controls	17.0 ± 1.3	0.4
		*PDE6C* HOMs	18.1 ± 1.3	
Ganglion cell layer	Temporal	WT controls	29.7 ± 1.7	0.2
		*PDE6C* HOMs	27.4 ± 1.7	
	Center	WT controls	NM	NM
		*PDE6C* HOMs	NM	
	Nasal	WT controls	37.9 ± 2.2	1.0
		*PDE6C* HOMs	38.0 ± 2.2	

No significant difference was noted in thickness between nerve fiber layer or ganglion cell layer at any location between *PDEC* HOMs and controls. Thickness measurements of the retinal layers were taken at three locations: 1.5 mm temporal to the foveal center, at the foveal center, and 1.5 mm nasal to the foveal center. NM, not measurable.

* *P* < 0.05, Student's *t*-test.

### Chromatic Pupillometry of Study Population

**Figure 4.**
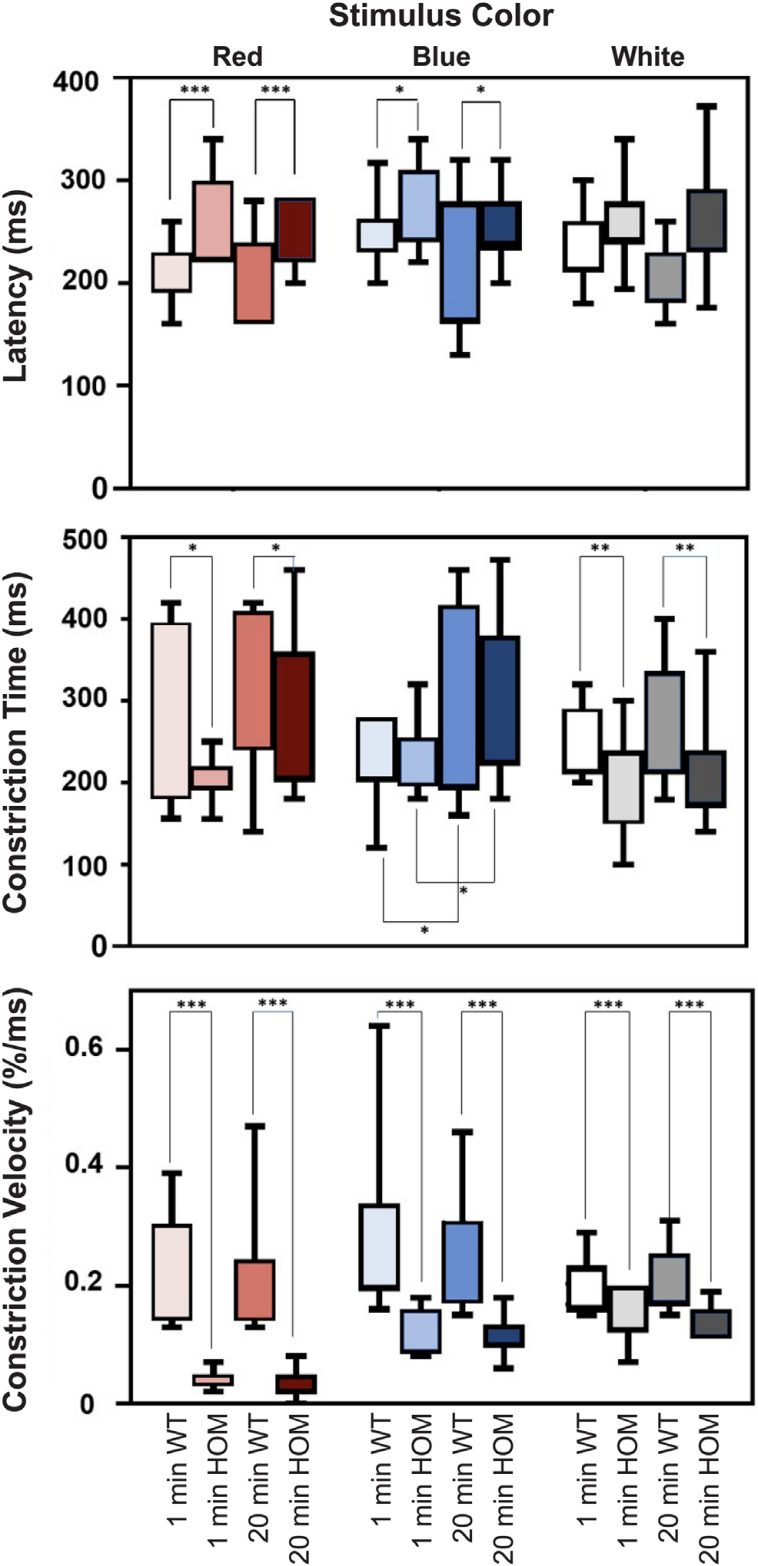
Box-and-whisker plot of mean pupil constriction latency, mean constriction time, and mean constriction velocity following red-, blue-, and white-light stimuli following 1- and 20-minute dark adaptation in WT control (W) and *PDE6C* HOM (H) rhesus macaques.

**Figure 5.**
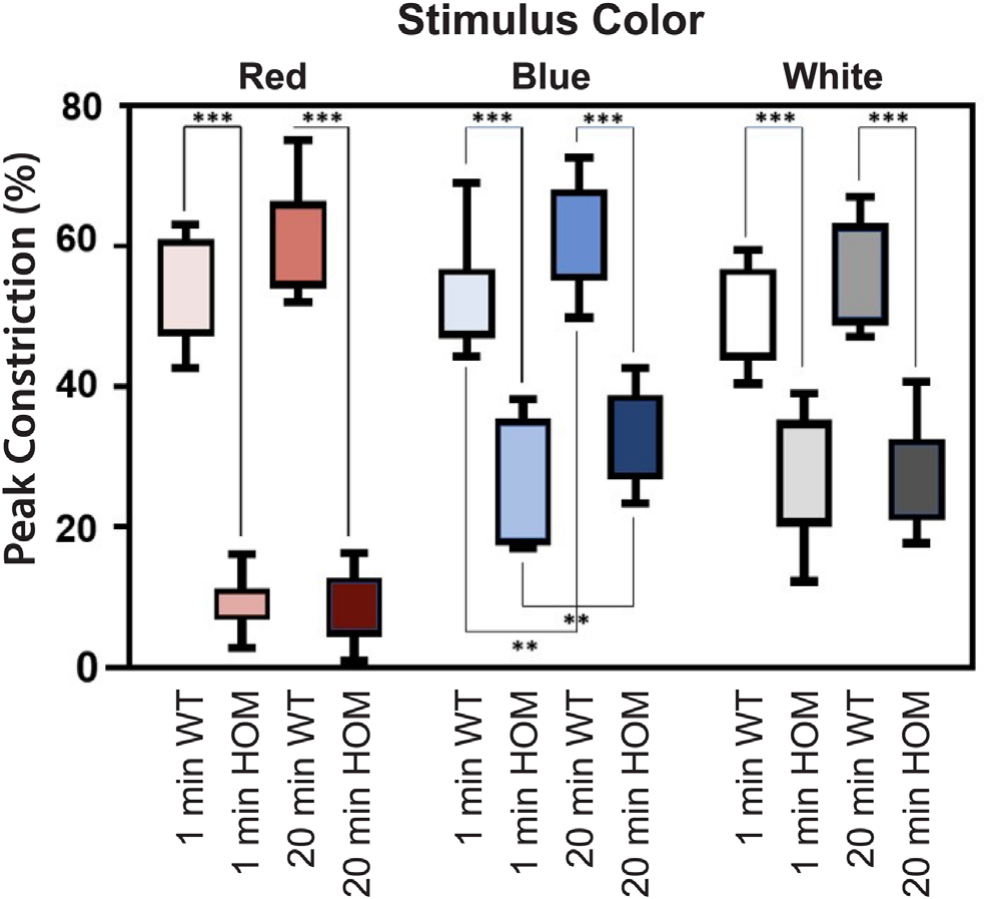
Box-and-whisker plot of mean peak pupil constriction following red-, blue-, and white-light stimuli following 1- and 20-minute dark adaptations in WT control and *PDE6C* HOM rhesus macaques.

**Figure 6.**
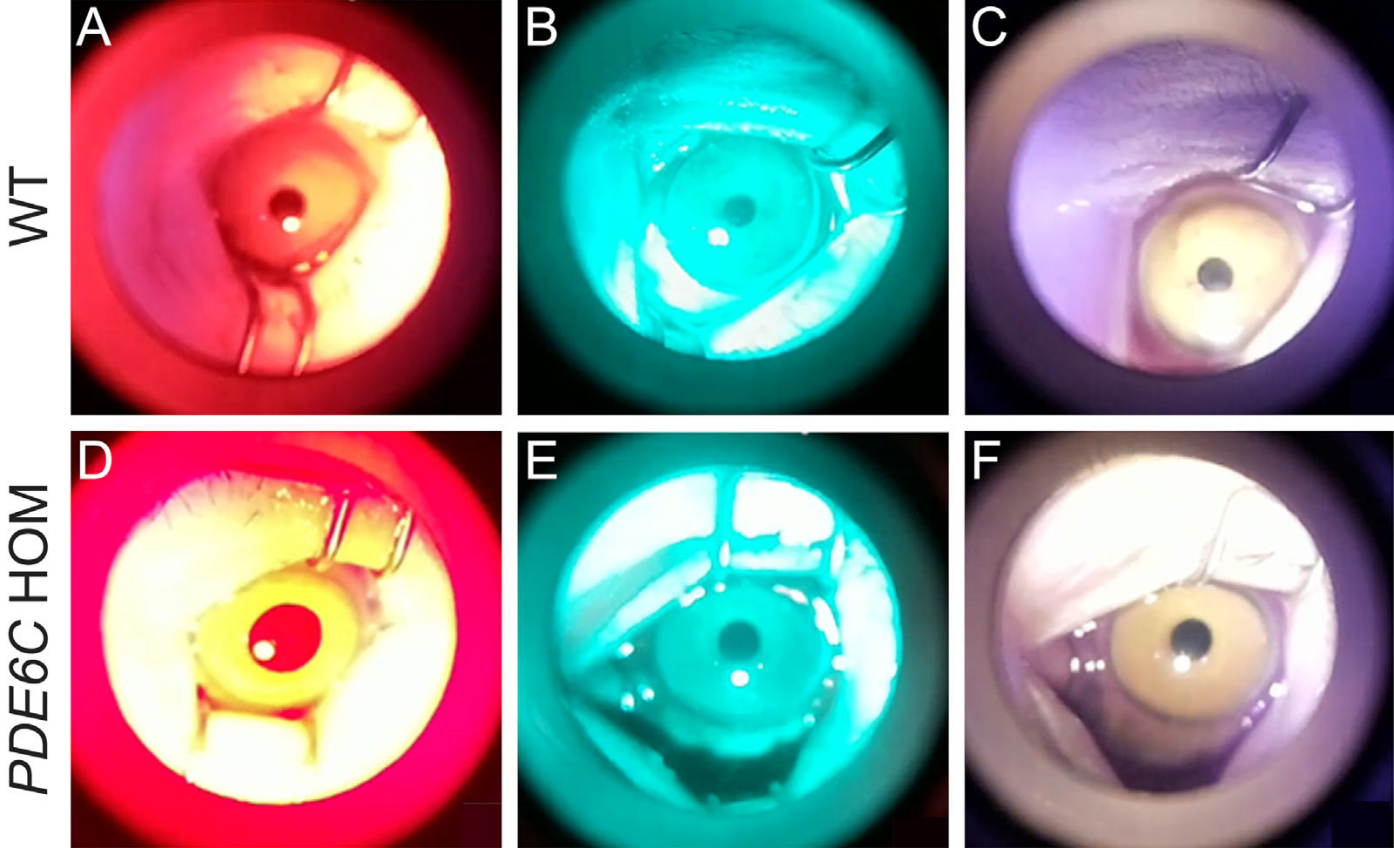
Pupillary images acquired from rhesus macaques using an infrared video retinoscopic attachment. Note the difference in maximum peak pupil constriction in WT rhesus macaques following 13 seconds of a red-light (**A**), blue-light (**B**), or white-light (**C**) stimulus compared to that in *PDE6C* HOM rhesus macaques following 13 seconds of a red-light (**D**), blue-light (**E**), or white-light (**F**) stimulus.

Resting pupil area was smaller in *PDE6C* HOM versus WT control rhesus macaques: 2.42 and 3.02 cm^2^, respectively (*P* = 0.02). Pupil constriction latency was longer in *PDE6C* HOMs versus WT controls for red-light (*P* = 0.0002) and blue-light (*P* = 0.04) stimulation but did not differ between groups following white-light stimulation (*P* = 0.20) (Fig. 4). Peak pupil constriction was less in *PDE6C* HOMs compared to WT controls with red-, blue-, and white-light stimulation (*P* < 0.0001) (Figs. 5, 6). Representative PLR waveforms are available for all light stimuli following both dark adaptation protocols (Supplementary Fig. S1). Pupil constriction time was shorter in *PDE6C* HOMs compared to controls with red-light (*P* = 0.04) and white-light (*P* = 0.003) stimulation but did not differ between groups following blue-light stimulation (*P* = 0.90) (Fig. 4). Pupil constriction velocity was slower in *PDE6C* HOMs versus controls with red-light (*P* < 0.0001), blue-light (*P* < 0.0001), and white-light (*P* = 0.0002) stimulation (Fig. 4).

No significant correlation was noted between the number of re-sedation events and resting pupil size (*r* = 0.4; *P* = 0.2) or peak pupil constriction (*r* = 0.3; *P* = 0.2). Dark adaptation time did correlate with peak pupil constriction following blue-light constriction time (*P* = 0.008), with the 20-minute protocol having a larger peak constriction compared to the 1-minute protocol at 47.2% versus 39.9% constriction, respectively (Fig. 5). Pupil constriction time following blue-light stimulation was also significantly different between dark adaptation protocols (*P* = 0.02) with the 1-minute protocol resulting in a slightly shorter constriction time compared to the 20-minute protocol at 233 and 300 ms, respectively (Fig. 4). Age and sex did not correlate with any significant differences among pupillometry measurements for red-, blue-, or white-light stimuli (Supplementary Table S1).

**Figure 7.**
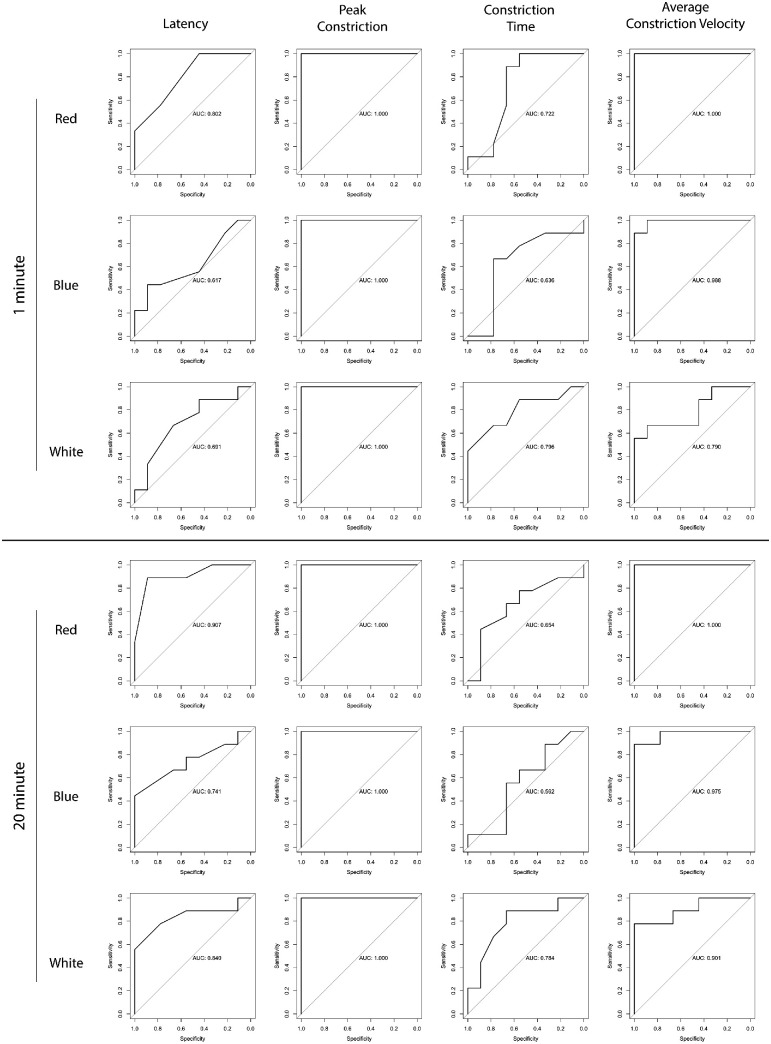
ROC curve analyses to evaluate the accuracy of the evaluated pupil parameters following red, blue, or white light stimulation with a 1- or 20-minute dark adaptation. An AUC > 0.8 was considered excellent, 0.7 to 0.8 was considered acceptable, and close to 0.5 was considered non-diagnostic.

Overall, chromatic pupillometry had high accuracy for determining phenotypic status, with red-light stimulation and peak constriction providing the most consistent accuracy. Seven of eight pupil parameters had excellent accuracy following red-light stimulation; constriction time following a 20-minute dark adaptation period was the only parameter to not show excellent accuracy with red-light stimulation (AUC = 0.65). Peak constriction consistently had excellent accuracy following all light stimuli with both a 1- and 20-minute dark adaptation. ROC curves are shown for all pupil parameters following the three light stimuli after 1- and 20-minute dark adaptations in Figure 7.

## Discussion

NHPs are vital research models for human ocular disease including IRDs due to their similarities in retinal anatomy, physiology, and genetics. Reduced feasibility and financial constraints associated with standardized ocular testing elevate the importance of developing new screening tools such as chromatic pupillometry for identifying naturally occurring ocular disease in these animals.

Chromatic pupillometry was hypothesized to detect abnormalities of various retinal cells involved in the PLR pathway in rhesus macaques with ACHM through the use of two different light wavelengths. This tool proved to be effective, with the most marked differences noted between *PDE6C* HOMs and WT controls following red-light stimuli, although significant differences were noted for blue- and white-light stimuli, as well. The red-light stimulus also proved to be the most accurate for phenotypic determination. As red-light–induced responses are a clinical approximation of primarily cone photoreceptor function, these results were expected given the cone dominant pathophysiology of PDE6C-associated cone disease. Similar reductions in pupil response to red-light stimuli have been noted in humans with cyclic nucleotide gated channel subunit alpha 3 (*CNGA3*)- and cyclic nucleotide gated channel subunit beta 3 (*CNGB3*)-mediated ACHM using chromatic Ganzfeld stimulation,^14^^,^^20^ with the latter study also noting similar changes in blue-light responses.^20^ In a study using chromatic pupillometry, reduced responses to red- and blue-light stimuli were noted in humans with *CNGA3*-mediated ACHM for cone-favoring photopic stimulation conditions with bright stimuli following light-adaptation, whereas increased responses to both stimuli were noted for rod-favoring scotopic stimulation characteristics with low-intensity stimuli in dark-adapted states.^16^ These novel results suggested an increased sensitivity to rod-favoring stimuli and evidence for disinhibition of ipRGCs in patients with CNGA3–ACHM. Unfortunately, none of these studies assessed pupil constriction velocity, latency, or constriction time for direct comparison to the current study.^14^^,^^16^^,^^20^ It is an open question whether or not rods and ipRGCs are hypersensitive in the absence of functioning cones in ACHM. This is one potential explanation for how pupil constriction is better preserved for blue compared to red stimuli in the context of absent cone function.

We hypothesize that the significant reduction in blue-light pupil responses in rhesus macaques with ACHM noted in the current study is most likely the result of absent blue cone contribution in *PDE6C* HOMs, although a true decrease in ipRGC function is also possible. Kardon et al.^14^ noted that dim blue stimuli (0.001 kcd/m^2^) could be used to approximate rod-mediated functions, whereas bright blue stimuli (0.1 kcd/m^2^) approximated ipRGC-mediated functions using chromatic pupillometry. The blue stimulus intensity used in the current study far exceeded that reportedly used by Kardon et al.,^14^ likely approximating both ipRGC-mediated and blue cone–mediated functions, suggesting that the reduction in blue stimulus pupillometry is most likely secondary to absent blue cone contribution to the PLR in *PDE6C* HOMs, although some contribution from rods cannot be ruled out. Additionally, the spectral sensitivity of blue (short-wavelength) cones has been confirmed to range from 381 to 600 nm in normal *Macaca* *fascicularis* NHPs, a close relative to rhesus macaques.^34^ The foremost theory is weakened, however, by the deep phenotyping of *PDE6C*-associated ACHM in humans, in which severe generalized cone system dysfunction was appreciated on full-field electroretinogram (ERG) with relative preservation of S-cone sensitivity on short-wavelength flash ERG.^35^ Perhaps in support of the theory of a true decrease in ipRGC function, Schroeder et al.^36^ noted the dependence of M4-type ipRGC on rods and cones for numerous response types, highlighting the intricate relationship and interdependence between the health of these retinal layers. Future studies are needed to investigate the physiologic function and microscopic features of ipRGCs in NHPs with ACHM to better determine the decrease in blue-light responses appreciated in the current study.

Chromatic pupillometry has been used to compare vertical pupil diameter constriction between Awassi sheep with *CNGA3*-associated ACHM and WT controls. Similar to the current study, a significant difference was noted between groups in peak constriction of vertical pupil diameter following red light stimulus. However, a disparate outcome was noted, with no significant difference in constriction between groups following blue- or white-light stimuli.^37^ The variable response to blue-light stimuli between sheep with *CNGA3-*associated ACHM in the aforementioned study and *PDE6C-*associated ACHM in the current study could be due to variation in genotype and clinical phenotype amongst ACHM-related genes. In humans, biallelic potential pathogenic variants in five of six ACHM-related genes, including *CNGA3* and *PDE6C*, were identified in 119 probands with genetic eye diseases; of these 119 probands, 62.2% had cone–rod dystrophy, whereas only 25.2% had ACHM.^38^

Despite intriguing differences noted in various pupil responses to blue and white light between groups, the current study shows that peak pupil constriction following red-light stimuli is the most advantageous for screening rhesus macaques with ACHM due to absent group overlap, large difference in means between groups, and freedom from reliance on calculations to determine relative changes in the field. We suspect that the relative miosis noted when comparing baseline pupil area in *PDE6C* HOM versus WT control rhesus macaques reflects the “pupillary constriction to darkness” response that has been reported in humans with poor vision secondary to congenital retinal disease such as congenital ACHM, stationary night blindness, and LCA.^39^ Although the methods of our study did not allow the investigation of variability in baseline pupil diameter between groups in light adaptation states, statistically smaller mean pupil diameter was noted in humans with *CNGA3*-linked ACHM compared to normal controls in both dark- and light-adaptation states.^16^ Lisowska et al.^16^ suggested that this difference was likely due to increased excitation of ipRGCs, which has been shown to result in pupil constriction.^40^ Suggested causes of elevated ipRGC excitation in patients with ACHM include photophobia and an absence of S-cones resulting in reduced cone-related inhibition of ipRGCs. These proposed mechanisms for a smaller mean resting pupil diameter in humans with ACHM may also be considered for the difference noted between groups in the current study. Though the smaller mean baseline pupil size is an interesting similarity between humans and NHPs with ACHM, this finding should not have affected our pupillometry results, as all calculations were based on relative pupil size changes from baseline.

In the current study, the dark-adaptation protocol was only a significant factor for two pupil response characteristics following blue light stimuli, suggesting adequate photoreceptor regeneration following a short 1-minute protocol. To the authors’ knowledge, this is the shortest dark-adaptation time tested in the context of IRD. To date, dark adaptation protocols employed for evaluating IRDs in humans have ranged from 10 to 20 minutes for ACHM,^14^^,^^16^^,^^20^ 2 to 10 minutes for retinitis pigmentosa,^15^^,^^17^^,^^18^ and 10 to 30 minutes for LCA.^15^^,^^19^^,^^20^ Although the findings of this study support the efficacy of a shortened dark adaptation for large group screening of IRDs in NHPs, further investigation is needed to determine if a shortened dark adaptation protocol has any utility for studying or screening IRDs in humans.

Limitations of this study include a limited sample size and single reference timepoint. Despite these limitations, the chromatic pupillometry responses measured between *PDE6C* HOMs and unaffected monkeys, as well as the overall accuracy of the test, argue for routine use of this instrument for IRD screening during NHP round-up events. Additionally, the results were not affected by age or sex, suggesting that screening a large NHP population with diverse genetic variation likely would retain a high signal-to-noise ratio with sufficient sensitivity and specificity to identify animals with suspected IRDs. Another consideration is that, although there are no known reports regarding the effects of the sedation protocol used in the current study on pupillary light reflexes in non-human primates, it is possible that this affected some or all of the pupil characteristics observed based on drug effects noted in humans.^41^^–^^43^ It is important to note, however, that sedation was kept consistent between and within treatment groups; therefore, any possible effects from the sedation on pupil characteristics should not have affected the significant differences identified. Additionally, no significant correlation was noted between the number of re-sedation events and resting pupil size or peak pupil constriction, suggesting limited effects of sedation on pupillary characteristics within the study, if present. Due to current sedation guidelines regarding the total sedation length and frequency of sedation episodes, testing could not be repeated on rhesus macaques by multiple observers to determine test repeatability and reproducibility.

## Conclusions

Chromatic pupillometry is a putative tool for screening NHPs for ACHM. A 1-minute dark adaptation protocol was sufficient to discern NHPs with and without ACHM, supporting its use during rapid screening events.

## Supplementary Material

Supplement 1
