# Supplementary material for: Chromatic Pupillometry as a Putative Screening Tool for Heritable Retinal Disease in Rhesus Macaques
Source: Transl Vis Sci Technol. 2023 Jun 22;12(6):13. doi: 10.1167/tvst.12.6.13 (PMC10289275; doi:10.1167/tvst.12.6.13)
Supplement: Supplement 1 [file tvst-12-6-13_s001.pdf]

SUPPLEMENTARY DATA

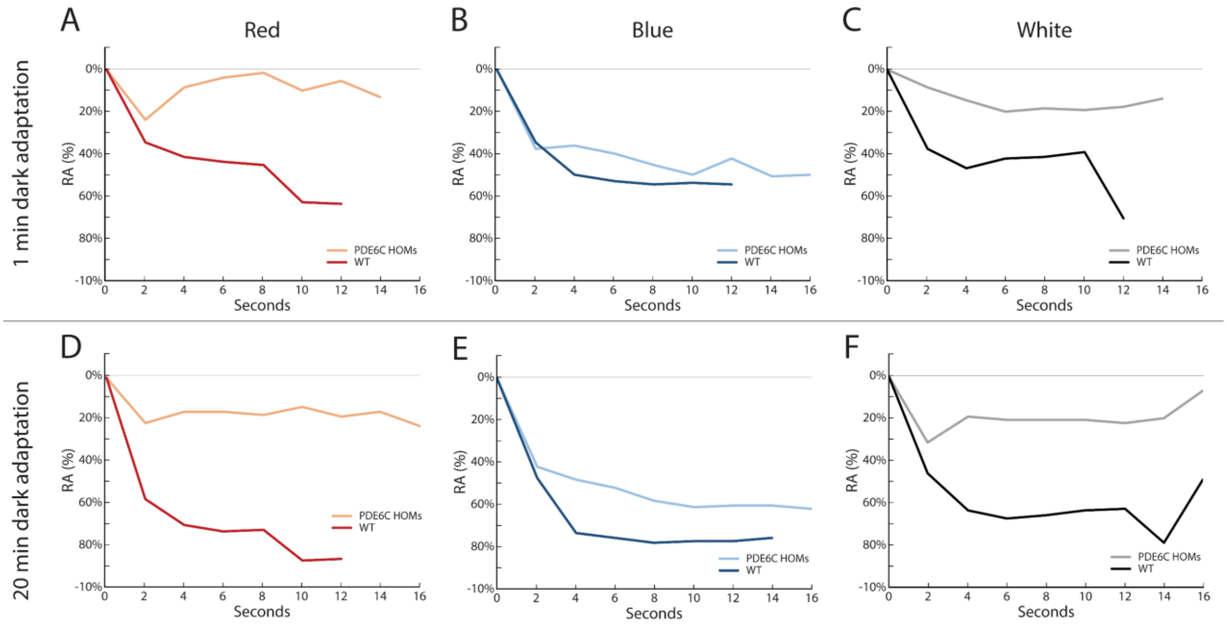

Figure S1. Pupillary response curves for *PDE6C* HOMs and WT rhesus macaques for red, blue, and white stimuli (stimulus duration 13 seconds from time point 0 seconds).

	Stimulus Color	Variable	Pearson's Coefficient	*P* value
Latency (ms)	Red	Age	0.1	0.6
		Sex	-0.05	0.8
	Blue	Age	0.09	0.7
		Sex	-0.3	0.2
	White	Age	0.2	0.4
		Sex	0.04	0.9
Peak Pupil Constriction (%)	Red	Age	0.03	0.9
		Sex	-0.006	1.0
	Blue	Age	0.03	0.9
		Sex	-0.05	0.8
	White	Age	-0.03	0.9
		Sex	0.004	1.0
Constriction time (ms)	Red	Age	0.3	0.3
		Sex	0.06	0.8
	Blue	Age	0.1	0.7
		Sex	-0.2	0.4
	White	Age	0.05	0.9
		Sex	-0.1	0.6
Constriction velocity (%/ms)	Red	Age	-0.2	0.6
		Sex	-0.03	0.9
	Blue	Age	-0.2	0.5
		Sex	-0.06	0.8
	White	Age	-0.1	0.6
		Sex	0.2	0.5

Table S1. Age and sex did not significantly impact chromatic pupillometry measurements following red-, blue-, and white-light stimuli in 9 WT and 9 *PDE6C* HOMs rhesus macaques.
